# Non-target GC–MS analyses of fecal VOCs in NASH-hepatocellular carcinoma model STAM mice

**DOI:** 10.1038/s41598-023-36091-7

**Published:** 2023-06-01

**Authors:** Mai Kato, Momoka Yamaguchi, Akira Ooka, Ryota Takahashi, Takuji Suzuki, Keita Onoda, Yuko Yoshikawa, Yuta Tsunematsu, Michio Sato, Yasukiyo Yoshioka, Miki Igarashi, Sumio Hayakawa, Kumiko Shoji, Yutaka Shoji, Tomohisa Ishikawa, Kenji Watanabe, Noriyuki Miyoshi

**Affiliations:** 1 Graduate School of Integrated Pharmaceutical and Nutritional Sciences, University of Shizuoka, 52-1 Yada, Suruga, Shizuoka, 422-8526 Japan; 2 Department of Food Science and Nutrition, Faculty of Human Life and Science, Doshisha Women’s College of Liberal Arts, Kyoto, Japan; 3 School of Veterinary Medicine, Faculty of Veterinary Science, Nippon Veterinary and Life Science University, Tokyo, Japan; 4 Advanced Clinical Research Center, Institute of Neurological Disorders, Kawasaki, Kanagawa Japan; 5 Department of Biochemistry and Molecular Biology, Graduate School of Medicine, Nippon Medical School, Tokyo, Japan; 6 Basic Nutrition, Kagawa Nutrition University, Saitama, Japan; 7 Department of Food Science and Nutrition, Shizuoka Eiwa Gakuin University Junior College, Shizuoka, Japan

**Keywords:** Biomarkers, Liver cancer, Non-alcoholic fatty liver disease, Inflammation, Microbiota

## Abstract

The increased incidence of obesity in the global population has increased the risk of several chronic inflammation-related diseases, including non-alcoholic steatohepatitis (NASH)-hepatocellular carcinoma (HCC). The progression from NASH to HCC involves a virus-independent liver carcinogenic mechanism; however, we currently lack effective treatment and prevention strategies. Several reports have suggested that fecal volatile organic compounds (VOCs) are strongly associated with NASH-HCC; therefore, we explored the biomarkers involved in its pathogenesis and progression. Fecal samples collected from control and NASH-HCC model STAM mice were subjected to headspace autosampler gas chromatography-electron ionization-mass spectrometry. Non-target profiling analysis identified diacetyl (2,3-butandione) as a fecal VOC that characterizes STAM mice. Although fecal diacetyl levels were correlated with the HCC in STAM mice, diacetyl is known as a cytotoxic/tissue-damaging compound rather than genotoxic or mutagenic; therefore, we examined the effect of bioactivity associated with NASH progression. We observed that diacetyl induced several pro-inflammatory molecules, including tumor necrosis factor-α, cyclooxygenase-2, monocyte chemoattractant protein-1, and transforming growth factor-β, in mouse macrophage RAW264.7 and Kupffer KPU5 cells. Additionally, we observed that diacetyl induced α-smooth muscle actin, one of the hallmarks of fibrosis, in an ex vivo cultured hepatic section, but not in in vitro hepatic stellate TWNT-1 cells. These results suggest that diacetyl would be a potential biomarker of fecal VOC in STAM mice, and its ability to trigger the macrophage-derived inflammation and fibrosis may partly contribute to NASH-HCC carcinogenesis.

## Introduction

Obesity is a leading cause of preventable deaths worldwide, with an estimated 2.8 million deaths annually^1^. Nevertheless, the prevalence of obesity is growing globally, approximately tripling since 1975 to 650 million in 2016^1^. Obesity increases the risk of several chronic inflammation-related diseases, including type 2 diabetes mellitus, fatty liver disease, hypertension, myocardial infarction, stroke, dementia, and cancer^2^. The incidence of liver cancer increases by 1.8-fold in obese people^3^. Hepatocellular carcinoma (HCC) is the most common type of liver cancer, the primary cause of which is viral hepatitis^4^. The pathology of viral hepatitis has been well studied, and therapeutic methods have been developed^5^. However, the development of HCC associated non-alcoholic fatty liver disease (NAFLD) and non-alcoholic steatohepatitis (NASH) has been increasing^6,7^. In principle, NAFLD is considered a liver lesion in metabolic syndrome owing to its association with obesity, diabetes, dyslipidemia, and hypertension as its etiologic background. NAFLD is classified into NAFL, which exhibits little disease progression, and NASH, which can progress to cirrhosis and liver cancer. The progression from hepatic steatosis and fibrosis to HCC was established in a virus-independent NAFLD-associated HCC model. Although the pathogenesis of NAFLD-associated HCC is reported to involve oxidative stress, inflammation, and DNA damage^6^, the molecular mechanisms of carcinogenesis have not yet been fully elucidated. Establishing effective prevention, diagnosis, and therapeutic strategies for NAFLD-HCC is crucial as its incidence is expected to increase in the future due to increase in obesity.

Metabolomic analyses have been conducted to identify biomarkers of NAFLD-HCC. Blood and urine samples collected from NAFLD-HCC model animals or patients were subjected to liquid chromatography-mass spectrometry (LC–MS), gas chromatography (GC)-MS, or nuclear magnetic resonance spectroscopy, and various organic compounds, including sugars, amino acids, lipids, nucleic acids, and their derivatives, were identified as candidate biomarkers^8^. In addition to blood and urine, fecal samples are also important biological specimens for exploring biomarkers of NAFLD-HCC. Fecal microbiota transplantation is a potential strategy to prevent the progression of NASH and improve the anticancer immune response, indicating that feces would be a promising biological source for identifying biomarkers^9^. Macrosmatic animals (dogs and mice) can distinguish the fecal odor of mice with transplanted HCC^10^. These reports suggest that fecal metabolites, especially volatile organic compounds (VOCs), can be potential biomarkers of NAFLD-HCC. Indeed, fecal VOC analysis was performed to evaluate the pathological features of gastrointestinal diseases^11–13^, including inflammatory bowel disease^14–16^, irritable bowel syndrome^14,17^, Crohn’s disease^18^, colorectal cancer^19,20^, and necrotizing enterocolitis^21,22^. Microflora and the host strongly interact with each other to maintain homeostasis; therefore, dysbiosis triggers both gastrointestinal and several chronic diseases. A human clinical study also reported that individuals with obesity and NAFLD display alterations in fecal microbiota and VOCs^23^. Representative VOCs in the intestinal tract include short-chain fatty acids (SCFAs). SCFAs produced by intestinal fermentation are consumed as an energy source. They have multiple functions, such as immune regulation, suppression of food intake, and enhancement of intestinal barrier function, which mediate anti-obesity, anti-diabetic, and anti-carcinogenic effects^24–29^. Therefore, fecal VOCs may be an important group of intestinal metabolites involved in the regulation of metabolic homeostasis. A recent study discussed the feasibility of exhaled breath VOCs as biomarkers for the diagnosis and monitoring of HCC progression and treatment response^30^. If VOCs are present throughout the body, they can be used as versatile non-invasive biomarkers.

In this study, we investigated fecal VOCs in NASH-HCC model STAM mice to identify potential biomarkers. NASH-HCC model STAM mice developed via subcutaneous injection of streptozotocin (STZ) on day 2 of birth following the high-fat diet (HFD) feeding exhibited sequential pathologies through the fatty liver, NASH, fibrosis, and HCC^31,32^. We performed a non-target headspace-autosampler (HSS)-GC-MS analysis and identified diacetyl (2,3-butandione) as a biomarker for characterizing STAM mice. Our results suggested that diacetyl triggers hepatic inflammation and fibrosis associated with liver carcinogenesis.

## Results

### Pathophysiological characteristics of STAM mice

**Figure 1:**
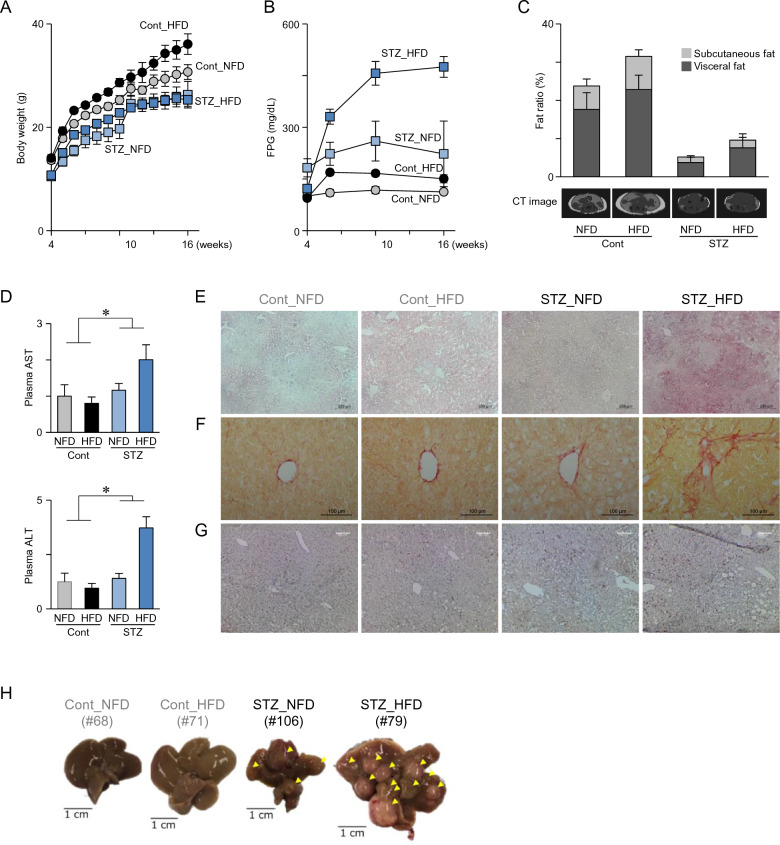
Pathophysiological characteristics of STAM mice. (**A**) Body weight and (**B**) fasting plasma glucose (FPG) levels in Cont_NFD, Cont_HFD, STZ_NFD, and STZ_HFD mice were measured (n = 5–17). (**C**) Computed tomography (CT) analysis of the fat ratio of mice at 16 weeks (n = 5–6). Fat ratio (%) for subcutaneous and visceral fat in a single scan at the level between the fourth and fifth lumbar vertebra was evaluated and summarized (n = 5–6). Typical CT scan images are shown at the bottom of the graph. (**D**) Plasma aspartate aminotransferase (AST) and alanine aminotransferase (ALT) in plasma collected on week 10 were determined (n = 5–6). *p < 0.05, two-way ANOVA. (**E**) Oil red O staining on week 6, (**F**) picrosirius red staining on week 10, and (**G**) immunohistochemistry (IHC) for macrophages (Mφ) on week 16 in Cont_NFD, Cont_HFD, STZ_NFD, and STZ_HFD were performed. (**H**) Representative macroscopic photographs of the liver at week 16. Arrowheads indicate hepatic tumors. Number in parentheses indicate animal IDs.

STAM mice (STZ_HFD) and three control mice (STZ_NFD, Cont_HFD, and Cont_NFD) were prepared by a single subcutaneous injection of STZ or saline (Cont) on the second day of birth and HFD or NFD-feeding. STZ groups have a background of late-stage type II diabetes; therefore, HFD feeding progresses to fatty liver disease, NASH, fibrosis, and liver cancer^31,32^. Body weight in the STZ groups were lower than those in the Cont groups, and HFD feeding increased the body weight in the Cont group (Fig. 1A). Higher levels of fasting plasma glucose (Fig. 1B) and lower levels of abdominal fat (Fig. 1C) were observed in the STZ groups, suggesting severe diabetic conditions. Additionally, significantly higher levels of plasma AST and ALT in the STZ groups indicated liver damage (Fig. 1D). STZ_HFD mice exhibited fatty liver (week 6), fibrosis (week 10), and macrophage infiltration (week 16) in histological analysis (Fig. 1E–G). Moreover, hepatic tumors were observed at week 16 in both STZ_NFD and STZ_HFD groups, with an incidence of 36% (4/11) and 85% (6/7), respectively (Fig. 1H). These results revealed that STAM mice (STZ_HFD group) developed NASH-associated liver cancer, despite some individual differences.

### GC–MS to determine the fecal VOC profile

**Figure 2:**
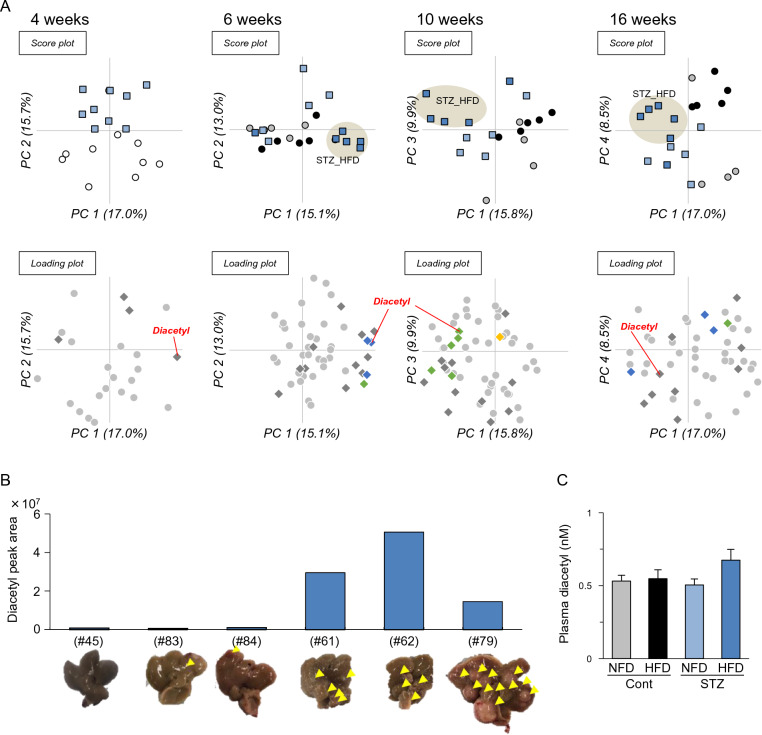
Headspace-autosampler-gas chromatography-mass spectrometry (HSS-GC-MS) analysis of fecal volatile organic compounds (VOCs). (**A**) The fecal VOC profile obtained via HSS-GC-MS analysis was subjected to principal component analysis (PCA) and analyzed by two-way ANOVA with multiple testing corrections using the Bonferroni family-wise error rate. Four experimental groups consisting of Cont_NFD, Cont_HFD, STZ_NFD, and STZ_HFD (n = 4–10) were distributed on PCA score plots. Ellipses drawn around the STZ-HFD group (STAM mice) on the PCA score plot do not have any statistical significance. On loading plots, diamond marks indicate compounds identified by EI spectrum search in NIST, but gray dots indicate not identified ones. Yellow and blue diamonds indicate compounds determined as significantly different by diet (NFD vs. HFD) and STZ injection, respectively (*p* < 0.05). Green diamonds indicate compounds determined as significant in an interaction between diet and streptozotocin (STZ) injection by two-way ANOVA. Diacetyl is indicated by red labels. (**B**) Individual levels of fecal diacetyl and hepatic tumor in STZ_HFD group (STAM) mice. Levels of fecal diacetyl were determined via HSS-GC-MS analysis of fecal samples collected from STAM mice (STZ_HFD group) on week 16. Hepatic tumors of corresponding individual liver collected on week 16 were indicated by yellow arrowheads. Number in parentheses indicate animal IDs. (**C**) Relative levels of plasma diacetyl determined by LC–MS. Mice plasma collected on week 16 were subjected to an extraction procedure, then injected into LC–MS (Means ± SE, n = 5–6).

To identify volatile biomarkers that reflect the pathophysiological alterations in STAM mice (STZ_HFD), fecal samples were analyzed using HSS-GC-MS. Raw data were processed to extract MS ion peaks, which were then integrated and filtered by frequency (60% presence in at least one group), resulting in MS ion peak integration to 24, 50, 58, and 50 entities at weeks 4, 6, 10, and 16, respectively (Supplementary Tables 1–4). Each integrated dataset was applied to principal component analysis (PCA) and two-way ANOVA. As shown in Fig. 2A, the STZ_HFD groups at weeks 6, 10, and 16 were clearly distinguished in PCA (score plot). Several entities were determined to have significant differences in diet (D) and STZ injection (S) by two-way ANOVA (Fig. 2A; Supplementary Tables 1–4). A strong contributor to HFD feeding seems to be nonanal. Nonanal was also detected in our previous study and was identified as a fecal VOC biomarker for HFD feeding in C57BL/6J and KK-*A*^*y*^ mice^33^. One of the most remarkable VOCs present in the STZ_HFD group was diacetyl (2,3-butanedione). Diacetyl did not exhibit any change at week 4 (before starting HFD feeding). However, there was a significant difference in its levels in the STZ and Cont groups at week 6 (*p* = 0.034) and a significant interaction between the two parameters, STZ, and diet (*p* = 0.023), at week 10 by two-way ANOVA (Supplementary Tables 2 and 3). Levels of diacetyl in STZ_HFD group were increased age-dependently with Pearson correlation coefficient of 0.484 (*p* = 0.0121, Supplementary Fig. 1). As shown in Fig. 2B and Supplementary Tables 5, a correlation between fecal diacetyl levels and tumor incidence, but not tumor diameter was observed at weeks 16 (*p* = 0.0028 with Pearson correlation coefficient 0.632). Fecal diacetyl levels in STZ_HFD were estimated at 2.6, 3.3, and 27.5 ppm by the external standard method. The plasma level of diacetyl in STZ_HFD was 0.67 ± 0.07 nM, which was slightly higher than that in other groups (Fig. 2C). These results suggest that diacetyl can be a plausible fecal biomarker for HCC incidence in STAM mice.

### Intestinal microbial composition and barrier function in STAM mice

**Figure 3:**
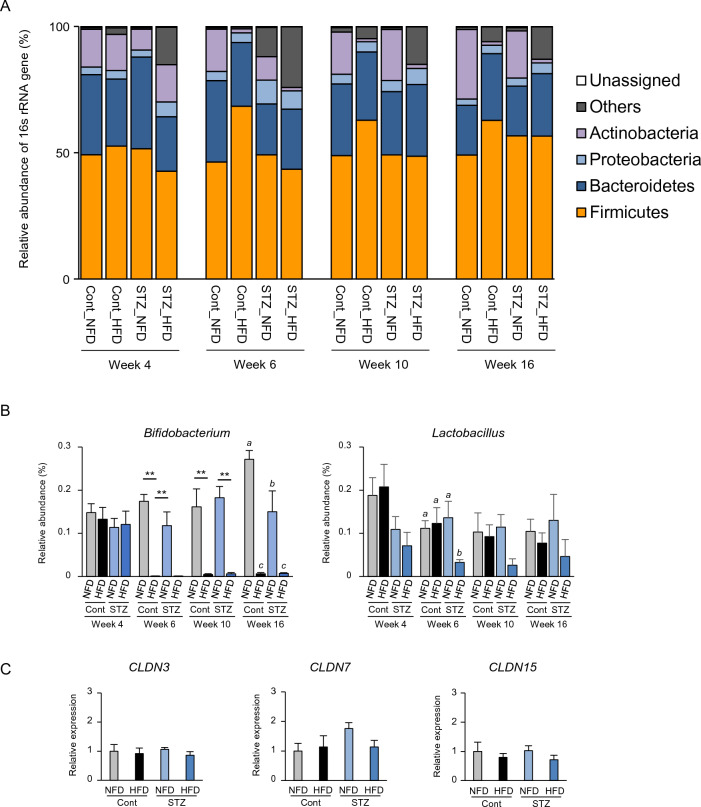
Intestinal microbial composition and gene expression of claudin family in the intestinal mucosa. (**A**) Microbial composition in mouse feces. First, 16 s rRNA sequencing was performed. Relative abundance (%) and taxonomic classification at the phylum level were analyzed using QIIME (n = 4–6). (**B**) Relative abundance (%) of *Bifidobacterium* and *Lactobacillus* are summarized (n = 4–6). Statistical significance was determined by two-way ANOVA. **p < 0.01. Bars designated with different letters are statistically different (*p* ≤ 0.05). (**C**) Gene expression levels of claudin-3, -7, and -15. Total RNA was prepared from large intestine mucosa collected at week 16, which were then subjected to reverse transcription-quantitative polymerase chain reaction (RT-qPCR; n = 5–6).

Next, 16s rRNA sequencing analysis revealed that the number of *Firmicutes* species was increased by HFD feeding in the Cont group but not in the STZ group (Fig. 3A). *Bifidobacterium* number was significantly reduced by HFD. *Lactobacillus* number was markedly decreased in STZ_HFD mice (Fig. 3B). Although HFD feeding induces metabolic endotoxemia associated with intestinal barrier leak^34^, the gene expression levels of *CLDN3* and *CLDN15* in the mucosa of the large intestine were slightly reduced in the HFD groups (Fig. 3C).

### Pro-inflammatory activities and liver fibrosis induced by diacetyl

**Figure 4:**
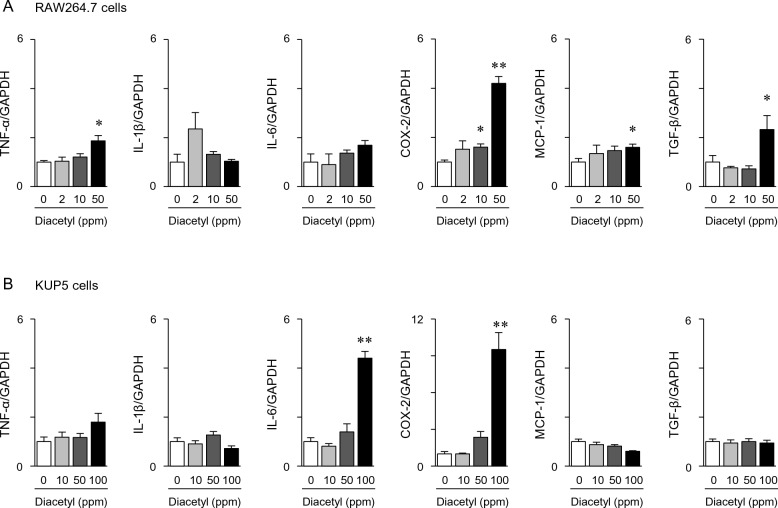
Effect of diacetyl on the pro-inflammatory gene expression in macrophage cells. Mouse macrophage RAW264.7 cells (**A**) and liver macrophage KPU5 cells (**B**) were exposed to diacetyl at the indicated concentration for six hours. Relative gene expression was analyzed via RT-qPCR. Values are represented as the mean ± SEM (n = 3–7). Statistical significance was determined by one-way ANOVA; *p < 0.05 and **p < 0.01 compared with control (0 ppm).

**Figure 5:**
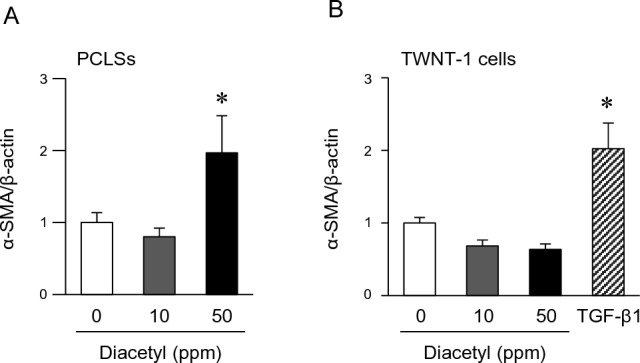
Effect of diacetyl on the expression levels of actin alpha 2 (*Acta2*) in ex vivo and in vitro. (**A**) RT-qPCR analysis of the mRNA expression of *Acta2* (α-SMA) in precision-cut liver slices (PCLSs) treated with or without diacetyl (10 and 50 ppm) for 5 days. Data are expressed as the mean ± SEM (n = 5). *p < 0.05, Holm’s multiple comparison test. (**B**) RT-qPCR analysis of the mRNA expression of *Acta2* (α-SMA) in human hepatic stellate TWNT-1 cells (passage 30) treated with or without diacetyl (10 and 50 ppm) and TGF-β1 (5 ng/mL) for 24 h. Data are expressed as the mean ± SEM (n = 4). *p < 0.05, Holm’s multiple comparison test.

Although diacetyl is toxic, especially in high doses, to the respiratory tract, including the nose, larynx, trachea, and lungs, it is not carcinogenic^35^. We observed infiltrated macrophages in the livers of the STZ_HFD group mice (Fig. 1E). Therefore, we evaluated the pro-inflammatory activities of diacetyl in mouse macrophage RAW264.7 and hepatic macrophage (Kupffer) KPU5 cells. Diacetyl dose-dependently upregulated TNF-α, COX-2, and MCP-1 levels in RAW264.7 and IL-6 and COX-2 levels in KPU5 cells (Fig. 4). We also observed that diacetyl induced TGF-β in RAW264.7 cells (Fig. 4), which is a master driver of tissue fibrosis^36,37^. We further evaluated the effects of diacetyl on liver fibrosis using an ex vivo mouse liver model. When mouse liver sections (precision-cut liver slices; PCLSs) were cultured with diacetyl, a hallmark of fibrosis, α-smooth muscle actin (α-SMA) expression was significantly induced (Fig. 5A). However, the induction of α-SMA was not observed when diacetyl was incubated in human hepatic stellate TWNT-1 cells (Fig. 5B) and mouse hepatic stellate cells were isolated from male ddY mice (Supplementary Fig. 2).

## Discussion

Recently, the incidence of NASH-HCC associated with diabetes and obesity has increased; therefore, early detection and treatment are essential. However, an effective diagnostic method has yet to be established. Analysis of volatile compounds in feces may be valuable as a new diagnostic method for systemic diseases. In this study, we performed non-target analyses of fecal VOCs in NASH-HCC model STAM mice using HSS-GC-MS, obtained fecal VOC profiles depending on the pathological process, and identified diacetyl (2,3-butanedione) as a promising biomarker for NASH-HCC.

Diacetyl is naturally present in various food items, including butter, milk, cheese, wine, and beer^38^. In addition, diacetyl has been used as a flavoring agent for many years. It has been approved for use as a generally regarded as safe (GRAS) product by the United States Food and Drug Administration. In food production, diacetyl is formed by chemical reactions, including lipid peroxidation, Maillard reactions, or microorganism fermentation^38^. Microorganisms produce diacetyl via acetolactate from pyruvate via a metabolic pathway^39^. In the animal body, there is another pathway in which pyruvate coupled with pyruvate dehydrogenase complex or its coenzyme thiamine pyrophosphate reacts with acetaldehyde to form acetoin, which is then converted to diacetyl^40^. Our results demonstrate that the fecal concentration of diacetyl was estimated to be about 500 times higher than that of plasma (Fig. 2C); therefore, the occurrence of diacetyl detected in the feces of STAM mice may be largely due to microorganism metabolism. Although diacetyl-producing bacterial species were not identified in the current study, 16s NGS sequencing analysis revealed that the numbers of *Bifidobacterium* and *Lactobacillus* were reduced, as previously reported in the intestinal bacterial composition of NASH^41,42^ (Fig. 3).

Although diacetyl is a natural ingredient in food and is not generally regarded as a health risk to consumers, airway (occupational) exposure can induce inflammation^43–45^. Even at low levels, chronic exposure of mice to diacetyl (100–400 ppm for 4 weeks) induces nasal and laryngeal toxicity and peribronchial and peribronchiolar lymphocytic inflammation^43^. Anderson et al*.* reported that exposure of human epithelial lung cancer A549 cells to diacetyl at 65 ppm induced TNF-α and IL-6 expression^46^. Our results also demonstrated that diacetyl significantly increased the mRNA levels of TNF-α and COX-2 in RAW264.7 cells (Fig. 4). These results suggest that diacetyl is a pro-inflammatory metabolite in STAM mice. COX-2 synthesizes various eicosanoids, including prostaglandins and thromboxanes, from arachidonic acid, and causes steatohepatitis by inducing lipid peroxidation and secretion of TNF-α and IL-6^47,48^. Furthermore, COX-2 is involved in NASH-liver carcinogenesis because its expression level increases in precancerous stage liver diseases, such as NASH and cirrhosis, inducing the subsequent onset of liver cancer^49,50^. In addition, diacetyl causes neutrophil infiltration into the lungs and enhances the permeability of epithelial cells, exhibiting pro-inflammatory activities^51,52^. The expression level of TGF-β was significantly increased by diacetyl treatment (Fig. 4). It significantly induced α-SMA expression in the cultured hepatic tissue sections (Fig. 5). These results suggest that diacetyl-induced inflammation and liver fibrosis via macrophage activation contribute to the progression of NASH-HCC in STAM mice. In contrast, diacetyl exhibited extremely weak mutagenic activity in the Ames test compared to α-dicarbonyl structural analogs, such as glyoxal and methylglyoxal^53^. In STAM mice, a high level of diacetyl is formed in the intestinal tract, which reaches the liver via the portal vein. STAM mice were chronically exposed to diacetyl. As diacetyl is toxic, long-term exposure may promote low-level chronic inflammation, a common risk factor for lifestyle-related diseases. Levels of diacetyl at week 6 was significantly increased in STZ injected mice, and which were further increased by the HFD feeding at week 10 (Fig. 2A and Supplementary Fig. 1). Diacetyl was one of the characteristic fecal metabolites of STAM mice at weeks 6–10, but not at week 4 (Fig. 2A). These results also suggested that long-term exposure of diacetyl may trigger an inflammatory response, which may be associated with NASH-HCC carcinogenesis.

NASH is characterized by steatosis, inflammation, and hepatocellular damage. NAFLD is a pathological condition in which fatty liver is recognized by tissue or imaging diagnosis, and other liver diseases, such as alcoholic liver injury, are excluded. Similar to alcoholic liver injury, increased intestinal permeability and elevated blood endotoxin levels are involved in NAFLD pathogenesis. Blood endotoxin levels are significantly elevated in patients with NAFLD, and intestinal permeability is significantly increased^54,55^. *Bifidobacteria* have been specifically associated with enhanced intestinal barrier function through the induction of tight junction proteins^56^. In STAM mice, the intestinal tight junctions did not appear to be disrupted, although the composition of gram-negative bacteria expressing endotoxin (LPS) was increased in STAM mice (Fig. 3). These findings suggest that diacetyl may not induce increased intestinal membrane permeability.

Here, we demonstrated that the occurrence of HCC was clearly correlated with fecal diacetyl levels in STAM (STZ-HFD group). Increased levels of diacetyl in STZ_HFD group was pathogenesis and progression dependent manner. The plasma levels of diacetyl determined using LC–MS were approximately 500 times lower than those in feces. Plasma samples used in this study were collected from the inferior vena cava of the abdomen, which circulates throughout the body. In contrast, portal vein blood contains microbial metabolites. Reid et al*.* reported that diacetyl and 2,3-pentandione levels were increased in the cecal content and portal vein blood of methionine- and choline-deficient diet-induced NASH model mice^52^. They also suggested that 2,3-pentanedione was cytotoxic and induced pro-inflammatory mediators in murine Kupffer cells. However, further studies are required to investigate the mechanism of diacetyl production and the correlation between NASH and HCC by comparing the diacetyl levels in venous and portal blood in NASH-HCC models.

## Materials and methods

### Chemicals

Diacetyl (2,3-butanedione) was purchased from Tokyo Chemical Industry Co. Ltd. STZ was purchased from Fujifilm Wako (Osaka, Japan). Dansyl hydrazine (DH),* p*-toluenesulfonic acid (pTsOH), and HistoDenz were purchased from Sigma-Aldrich (St. Louis, MO, USA). PRONASE protease from *Streptomyces griseus* (Merck-Millipore, MA, USA), and collagenase (Yakult, Tokyo, Japan) were also used.

### Animal experiments using the NASH-HCC model

Animal experiments were approved by the Animal Ethics Committee of the University of Shizuoka (approval number 175171) and performed according to the ARRIVE guidelines. To establish STAM mice, male C57BL/6J mice were subjected to a single subcutaneous injection of 200 μg STZ on the second day after birth and fed a high-fat diet (HFD32; CLEA Japan, Tokyo, Japan) ad libitum from weaning period (four weeks of age). STAM mice were grouped as STZ_HFD. Additionally, three control groups of mice were created via injection with STZ or saline on day 2 of birth and administered a normal fat diet (AIN76) or HFD, which were then grouped as Cont_NFD, Cont_HFD, and STZ_NFD, respectively. All animals were housed individually in plastic cages after weaning and had free access to drinking water under controlled conditions of humidity (55 ± 5%), light (12/12-h light/dark cycle), and temperature (23 ± 1 °C). Abdominal CT images were obtained using COSMOScan FX (Rigaku, Tokyo, Japan). The mice were sacrificed under deep anesthesia with inhalation of isoflurane at weeks 4, 6, 10, and 16 after 16 h of fasting. Blood samples were obtained from the inferior vena cava to determine the blood glucose, triglyceride, aspartate aminotransferase (AST), and alanine aminotransferase (ALT) levels using ACCU-Chek Aviva (Roche, Indianapolis, IN, USA), triglyceride E test (Wako Pure Chemical, Japan), AST Activity Assay kit (BioVision, CA, USA), and ALT Activity Assay kit (BioVision), respectively. Liver tissues were macroscopically inspected for the presence of tumor, and examined using hematoxylin–eosin staining, Oil red O staining, and immunohistochemistry using biotin anti-mouse F4/80 antibody (BioLegend, SanDiego, CA, USA). Aliquots (100 mg) of feces were immediately subjected to HSS-GC-MS.

### Cell culture

Mouse macrophage RAW264.7 cells (RIKEN Cell Bank, Ibaraki, Japan) and human hepatic stellate TWNT-1 cells (JCRB Cell Bank, Tokyo, Japan) in 10% FBS/DMEM, and mouse liver macrophage (Kupffer) KPU5 cells (RIKEN Cell Bank, Ibaraki, Japan) in 10% FBS/DMEM supplemented with 10 µg/ml bovine insulin and 250 µM monothioglycerol were maintained and grown in an atmosphere of 95% air and 5% CO_2_ at 37 °C.

### Quantitative reverse transcription-polymerase chain reaction (qRT-PCR)

Total RNA was extracted using the TRIzol reagent (Invitrogen) and converted into cDNA using the PrimeScript RT Master Mix (TaKaRa). To quantitatively estimate the expression level of each gene, quantitative PCR was performed using gene-specific primers, cDNA, and SYBR Premix (TaKaRa). The sequences of the PCR primer pairs were as follows:* GAPDH*, 5′-AAA ATG GTG AAG GTC GGT GTG-3′ and 5′-AAT GAA GGG GTC GTT GAT GG-3′; tumor necrosis factor (*TNF*)-*α*, 5′-GAT TAT GGC TCA GGG TCC AA-3′ and 5′-CCC AGC ATC TTG TGT TTC TG-3′; interleukin (*IL*)*-1β*, 5′-TCT TCC TAA AAG TAT GGG CTG GA-3′ and 5′-AAA AGG GAG CTC CTT AAC ATG C-3′; *IL-6*, 5′-AGT CCT TCC TAC CCC AAT TTC C-3′ and 5′-TTG GAT GGT CTT GGT CCT TAG C-3′; cyclooxygenase 2 (*COX-2*), 5′-GGA GGC GAA GTG GGT TTT AAG-3′ and 5′-TTG ATG GTG GCT GTT TTG GTA G-3′; monocyte chemoattractant protein-1 (*MCP-1*), 5′-CCA CTC ACC TGC TGC TAC TCA T-3′ and 5′-TGG TGA TCC TCT TGT AGC TCT CC-3′; transforming growth factor (*TGF*)*-β*, 5′-GCC TCG CTG TCT GAG AGT TC-3′ and 5′-CAC TGT GTG GGT GGG ATG TA-3′; claudin 3 (*CLDN3*), 5′-GGG AGT GCT TTT CCT GTT GG-3′ and 5′-AAT CCC TGA TGA TGG TGT TGG-3′; *CLDN7*, 5′-GGG GAG ATG ACA AAG CGA AG-3′ and 5′-CAA GAC CTG CCA CAA TGA AAA C-3′; *CLDN15*, 5′-ACT GCT GAT GCT GGG GTT G-3′ and 5′-AAA GAT GGT GTT GGT GGT GAT G-3′; *Acta2*, 5′-GAC GTA CAA CTG GTA TTG TG-3′ and 5′-TCA GGA TCT TCA TGA GGT AG-3′; *Actb*, 5′-GCC ATG GAT GAC GAT ATC GC-3′ and 5′-CCC AGT TGG TAA CAA TGC CA-3′.

### NGS 16s sequencing

The microbial composition of feces was analyzed as described in our previous studies^33,57^. DNA in feces was extracted (Stool DNA Isolation Kit), and the V3-V4 variable region of 16s rRNA was amplified using a specific primer (5′-TCG TCG GCA GCG TCA GAT GTG TAT AAG AGA CAG CCT ACG GGN GGC WGC AG-3′ and 5′-GTC TCG TGG GCT CGG AGA TGT GTA TAA GAG ACA GGA CTA CHV GGG TAT CTA ATC C-3′). The quantified amplicons were tagged with barcodes and sequenced using MiSeq (Illumina, San Diego, CA, USA). The raw reads are were deposited in DDBJ under DRA accession number DRA015492, which is available from DDBJ search (https://ddbj.nig.ac.jp/search). Data were analyzed using Nephele (National Institutes of Health, Bethesda, MD, USA) and QIIME^58^.

### HSS-electron ionization (EI)-GC-MS

Fecal VOCs were analyzed using HSS-GC-MS consisting of an Agilent 7697 headspace sampler, an Agilent 7890 gas chromatograph, and an Agilent 5975 mass spectrometer, as described in our previous study^33,59^. Fecal samples (100 mg) in a barotolerant vial were heated in HSS at 100 °C for 60 min, then injected (50 mL/min for 1 min) with a split ratio (5:1) to GC through the inlet maintained at 250 °C. VOCs in samples were separated using a DB-WAX column (30 m, 0.25 mm, 0.25 µm) at an initial temperature of 35 °C for 1 min, which was increased by 5 °C/min to 120 °C, and maintained at 250 °C for 10 min. Helium was used as the carrier gas at 1.1 mi/min constant flow. Ion source (EI) and quadrupole were maintained at 230 and 150 °C, respectively. VOCs detected at *m/z* 14–500 were monitored. MS peak data from HSS-GC-MS analyses were subjected to AMDIS software analysis for peak detection and integration and Mass Profiler Professional software (Agilent Technology) for data processing and principal component analysis. The levels of diacetyl in the feces detected by GC–MS were estimated using standard external methods.

### LC–MS to determine fecal diacetyl levels

Plasma levels of diacetyl were determined using LC–MS, as previously reported, with slight modifications^57^. Briefly, 20 µL of plasma was added to a tube containing 80 µL ultrapure water and 200 µL CHCl_3_/MeOH (2:1). The tube was vortexed, centrifuged, and the organic phase was harvested in new tubes. The residual aqueous phase was added to 200 µL of CHCl_3_/MeOH (2:1), vortexed, and centrifuged, and the harvested organic phase was combined. The organic phase was mixed with 100 µL of acetonitrile containing 50 µg DH and 10 µg *p*-TsOH, vortexed, and incubated for 4 h in the dark. The sample was dried by evaporation, dissolved in 200 µL acetonitrile, and subjected to LC–MS analyses. LC–MS analyses were performed using an Agilent 1290 series HPLC coupled with a G6410B triple quadrupole tandem mass spectrometer. HPLC separation was performed using a ZORBAX Eclipse Plus C18 column (1.8 µm, 50 × 2.1 mm; Agilent Technologies) at 40 °C. The mobile phases A and B were ultrapure water (0.1% formic acid) and acetonitrile (0.1% formic acid), respectively. Diacetyl-DH was detected by monitoring its transition in the ESI-positive MRM mode 294.1 > 236. Under these conditions, the recovery rate was 81% (n = 3).

### Precision cut liver slices (PCLSs)

Liver tissue from mice was glued to the vibratome mounting stage using Aron Alpha (Toagosei, Tokyo, Japan) and placed on ice. Liver tissue was submerged in the media chamber containing cold Krebs–Henseleit buffer (118 mM NaCl, 4.7 mM KCl, 1.2 mM MgSO_4_, 1.25 mM CaCl_2_, 1.2 mM KH_2_PO_4_, 11 mM d-glucose, 25 mM NaHCO_3_) and cut using a Leica VT1200S vibrating blade microtome (Leica Biosystems, Milton Keynes, UK) at a speed 1.0 mm/s, amplitude of 1.0 mm, and thickness of 250 μm. PCLSs were transferred into standard 6-well plates, and cultured in the Roswell Park Memorial Institute-1640 medium (Sigma-Aldrich) supplemented with penicillin/streptomycin, 10% fetal bovine serum (Gibco, Life Technologies), and diacetyl (0–10 ppm) at 37 °C in a 5% CO_2_ atmosphere for 5 days. The medium was changed daily. Total RNA was extracted from PCLSs using the NucleoSpin RNA kit (MACHEREY–NAGEL, Duren, Germany).

### Statistical analysis

All data are presented as the mean ± SE. All statistical analyses were performed using EZR (Saitama Medical Center, Jichi Medical University), a graphical user interface for R (The R Foundation for Statistical Computing). Statistical analyses of the data were performed using one-way and two-way ANOVA. Differences were considered statistically significant at *p* < 0.05.

### Ethics declaration

All animal experiment methods were performed in accordance with the relevant guidelines and regulations. Authors complied with all the relevant guidelines and regulations and complied with ARRIVE guidelines.

## Supplementary Information

Supplementary Figures.

Supplementary Table S1.

Supplementary Table S2.

Supplementary Table S3.

Supplementary Table S4.

Supplementary Table S5.

## Data Availability

All raw sequences have been deposited to the DDBJ with accession number DRA015492 (https://ddbj.nig.ac.jp/search?query=%22DRA015492%22).

## References

[1] WHO. Obesity and overweight. *Fact sheet* (2018).

[2] Blüher M (2019). Obesity: Global epidemiology and pathogenesis. Nat. Rev. Endocrinol..

[3] Lauby-Secretan B (2016). Body fatness and cancer-viewpoint of the IARC Working Group. N. Engl. J. Med..

[4] Suresh D, Srinivas AN, Kumar DP (2020). Etiology of hepatocellular carcinoma: Special focus on fatty liver disease. Front. Oncol..

[5] Hoofnagle JH, di Bisceglie AM (1997). The treatment of chronic viral hepatitis. N. Engl. J. Med..

[6] Anstee QM, Reeves HL, Kotsiliti E, Govaere O, Heikenwalder M (2019). From NASH to HCC: Current concepts and future challenges. Nat. Rev. Gastroenterol. Hepatol..

[7] Negro F (2020). Natural history of NASH and HCC. Liver Int..

[8] Safaei A (2016). Metabolomic analysis of human cirrhosis, hepatocellular carcinoma, non-alcoholic fatty liver disease and non-alcoholic steatohepatitis diseases. Gastroenterol. Hepatol. from bed to bench.

[9] Delaune V (2018). Fecal microbiota transplantation: A promising strategy in preventing the progression of non-alcoholic steatohepatitis and improving the anti-cancer immune response. Expert Opin. Biol. Ther..

[10] Rodionova, E. I. *et al.* Detection of volatile organic compounds associated with hepatocellular carcinoma by macrosmatic animals: Approaches to the search for new tumor markers. *Izv. Akad. Nauk. Seriia Biol.* 293–301. 26349235

[11] Chan DK, Leggett CL, Wang KK (2016). Diagnosing gastrointestinal illnesses using fecal headspace volatile organic compounds. World J. Gastroenterol..

[12] Walton C (2013). Analysis of volatile organic compounds of bacterial origin in chronic gastrointestinal diseases. Inflamm. Bowel Dis..

[13] Garner CE (2007). Volatile organic compounds from feces and their potential for diagnosis of gastrointestinal disease. FASEB J..

[14] Bosch S (2018). Differentiation between pediatric irritable bowel syndrome and inflammatory Bowel disease based on fecal scent: Proof of principle study. Inflamm. Bowel Dis..

[15] Probert CSJ, Reade S, Ahmed I (2014). Fecal volatile organic compounds: A novel, cheaper method of diagnosing inflammatory bowel disease?. Expert Rev. Clin. Immunol..

[16] Probert CSJ (2011). Role of faecal gas analysis for the diagnosis of IBD. Biochem. Soc. Trans..

[17] Rossi M (2018). Volatile organic compounds in feces associate with response to dietary intervention in patients with irritable bowel syndrome. Clin. Gastroenterol. Hepatol..

[18] Kennedy NA (2018). The impact of NOD2 variants on fecal microbiota in Crohn’s disease and controls without gastrointestinal disease. Inflamm. Bowel Dis..

[19] Di Lena M, Porcelli F, Altomare DF (2016). Volatile organic compounds as new biomarkers for colorectal cancer: A review. Colorectal Dis..

[20] de Meij TG (2014). Electronic nose can discriminate colorectal carcinoma and advanced adenomas by fecal volatile biomarker analysis: Proof of principle study. Int. J. cancer.

[21] de Meij TGJ (2015). Early detection of necrotizing enterocolitis by fecal volatile organic compounds analysis. J. Pediatr..

[22] Garner CE (2009). Analysis of faecal volatile organic compounds in preterm infants who develop necrotising enterocolitis: A pilot study. J. Pediatr. Gastroenterol. Nutr..

[23] Boursier J, Rawls JF, Diehl AM (2013). Obese humans with nonalcoholic fatty liver disease display alterations in fecal microbiota and volatile organic compounds. Clin. Gastroenterol. Hepatol..

[24] Goswami C, Iwasaki Y, Yada T (2018). Short-chain fatty acids suppress food intake by activating vagal afferent neurons. J. Nutr. Biochem..

[25] Khan MT, Nieuwdorp M, Bäckhed F (2014). Microbial modulation of insulin sensitivity. Cell Metab..

[26] De Vadder F (2014). Microbiota-generated metabolites promote metabolic benefits via gut-brain neural circuits. Cell.

[27] Chambers ES (2015). Effects of targeted delivery of propionate to the human colon on appetite regulation, body weight maintenance and adiposity in overweight adults. Gut.

[28] Tang C (2015). Loss of FFA2 and FFA3 increases insulin secretion and improves glucose tolerance in type 2 diabetes. Nat. Med..

[29] McNelis JC (2015). GPR43 potentiates β-cell function in obesity. Diabetes.

[30] Sukaram T (2022). Exhaled volatile organic compounds for diagnosis of hepatocellular carcinoma. Sci. Rep..

[31] Fujii M (2013). A murine model for non-alcoholic steatohepatitis showing evidence of association between diabetes and hepatocellular carcinoma. Med. Mol. Morphol..

[32] Takahashi Y, Soejima Y, Fukusato T (2012). Animal models of nonalcoholic fatty liver disease/nonalcoholic steatohepatitis. World J. Gastroenterol..

[33] Uchikawa M (2020). Elevated levels of proinflammatory volatile metabolites in feces of high fat diet fed KK-Ay mice. Sci. Rep..

[34] Ahmad R, Rah B, Bastola D, Dhawan P, Singh AB (2017). Obesity-induces organ and tissue specific tight junction restructuring and barrier deregulation by claudin switching. Sci. Rep..

[35] *NTP Technical Report on the Toxicology and Carcinogenesis Studies of 2,3-Butanedione (CAS NO. 431-03-8) in Wistar Han [Crl:WI (Han)] Rats and B6C3F1/N Mice (Inhalation Studies)* https://ntp.niehs.nih.gov/go/tr593abs (2018) 10.22427/NTP-TR-593. 10.22427/NTP-TR-593 PMC8039897 33562897

[36] Fabregat I (2016). TGF-β signalling and liver disease. FEBS J..

[37] Fabregat I, Caballero-Díaz D (2018). Transforming growth factor-β-induced cell plasticity in liver fibrosis and hepatocarcinogenesis. Front. Oncol..

[38] Shibamoto T (2014). Diacetyl: Occurrence, analysis, and toxicity. J. Agric. Food Chem..

[39] Hara T, Matsui H, Shimizu H (2014). Suppression of microbial metabolic pathways inhibits the generation of the human body odor component diacetyl by *Staphylococcus* spp. PLoS ONE.

[40] Otsuka M, Mine T, Ohuchi K, Ohmori S (1996). A detoxication route for acetaldehyde: Metabolism of diacetyl, acetoin, and 2,3-butanediol in liver homogenate and perfused liver of rats. J. Biochem..

[41] Siebler J, Galle PR, Weber MM (2008). The gut-liver-axis: Endotoxemia, inflammation, insulin resistance and NASH. J. Hepatol..

[42] Okubo H (2013). *Lactobacillus casei* strain Shirota protects against nonalcoholic steatohepatitis development in a rodent model. Am. J. Physiol. Gastrointest. Liver Physiol..

[43] Morgan DL, Flake GP, Kirby PJ, Palmer SM (2008). Respiratory toxicity of diacetyl in C57BL/6 mice. Toxicol. Sci..

[44] Larsen ST, Alarie Y, Hammer M, Nielsen GD (2009). Acute airway effects of diacetyl in mice. Inhal. Toxicol..

[45] Starek-Swiechowicz B, Starek A (2014). Diacetyl exposure as a pneumotoxic factor: A review. Rocz. Panstw. Zakl. Hig..

[46] Anderson SE, Jackson LG, Franko J, Wells JR (2010). Evaluation of dicarbonyls generated in a simulated indoor air environment using an in vitro exposure system. Toxicol. Sci..

[47] Bayomi EA (2015). Cyclooxygenase-2 expression is associated with elevated aspartate aminotransferase level in hepatocellular carcinoma. J. Cancer Res. Ther..

[48] Yu J (2006). COX-2 induction in mice with experimental nutritional steatohepatitis: Role as pro-inflammatory mediator. Hepatology.

[49] Loo TM (2017). Gut microbiota promotes obesity-associated liver cancer through PGE2-mediated suppression of antitumor immunity. Cancer Discov..

[50] Luo Y (2019). Berberine prevents non-alcoholic steatohepatitis-derived hepatocellular carcinoma by inhibiting inflammation and angiogenesis in mice. Am. J. Transl. Res..

[51] Yoshimine Y (2015). Hepatic expression of the Sptlc3 subunit of serine palmitoyltransferase is associated with the development of hepatocellular carcinoma in a mouse model of nonalcoholic steatohepatitis. Oncol. Rep..

[52] Reid DT (2016). Unique microbial-derived volatile organic compounds in portal venous circulation in murine non-alcoholic fatty liver disease. Biochim. Biophys. Acta.

[53] Dorado L, Ruis Montoya MR, Rodríguez Mellado JM (1992). A contribution to the study of the structure-mutagenicity relationship for alpha-dicarbonyl compounds using the Ames test. Mutat. Res..

[54] Harte AL (2010). Elevated endotoxin levels in non-alcoholic fatty liver disease. J. Inflamm..

[55] Kessoku T (2021). Endotoxins and non-alcoholic fatty liver disease. Front. Endocrinol..

[56] Cani PD (2009). Changes in gut microbiota control inflammation in obese mice through a mechanism involving GLP-2-driven improvement of gut permeability. Gut.

[57] Sanada S (2020). Intestinal microbial metabolite stercobilin involvement in the chronic inflammation of ob/ob mice. Sci. Rep..

[58] Caporaso JG (2010). QIIME allows analysis of high-throughput community sequencing data. Nat. Methods.

[59] Kato Y (2021). Methylglyoxal binds to amines in honey matrix and 2′-methoxyacetophenone is released in gaseous form into the headspace on the heating of manuka honey. Food Chem..

