# Supplementary material for: Non-target GC–MS analyses of fecal VOCs in NASH-hepatocellular carcinoma model STAM mice
Source: Sci Rep. 2023 Jun 1;13:8924. doi: 10.1038/s41598-023-36091-7 (PMC10235110; doi:10.1038/s41598-023-36091-7)
Supplement: Supplementary file 1 — Supplementary Figures. [file 41598_2023_36091_MOESM1_ESM.pdf]

Supplementary figure 1

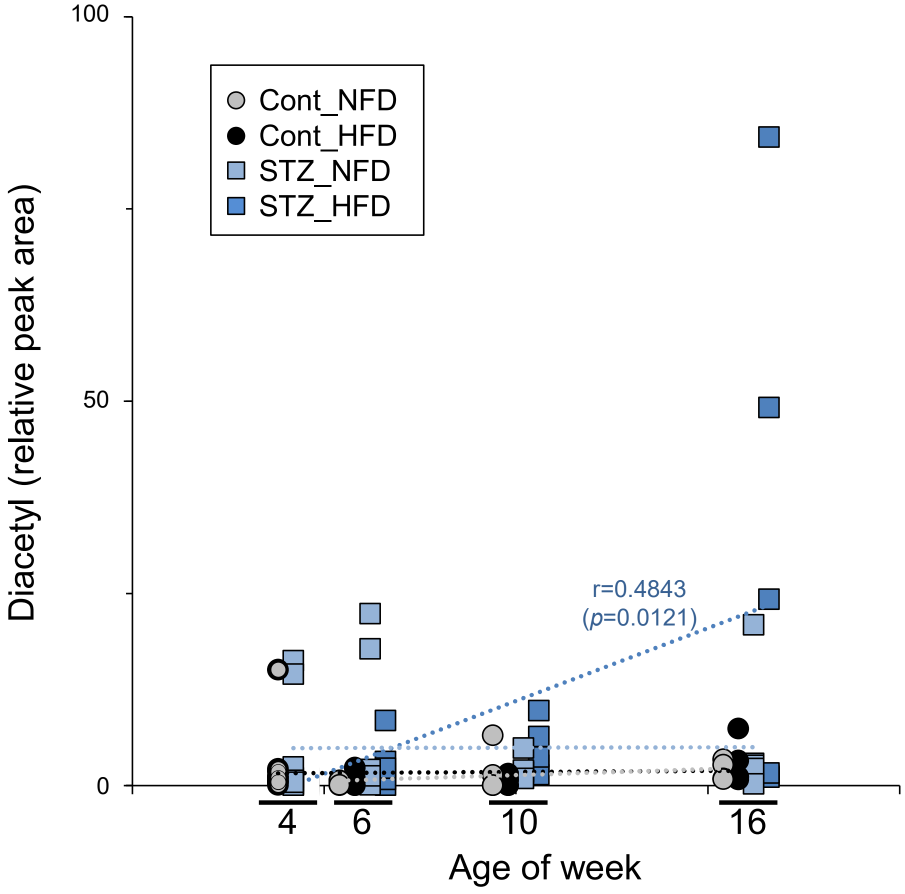

Fig. S1 Relative levels of fecal diacetyl

Fecal samples collected from Cont_NFD, Cont_HFD, STZ_NFD, and STZ_HFD (n = 4–10) were subjected to GC-MS to determine levels of diacetyl as described in Materials and Methods section. Peak areas for diacetyl were summarized. Trend lines for each group are indicated by dashes. Pearson correlation coefficient of STZ_HFD group was determined using EZR (Saitama Medical Center, Jichi Medical University), a graphical user interface for R (The R Foundation for Statistical Computing).

Supplementary figure 2

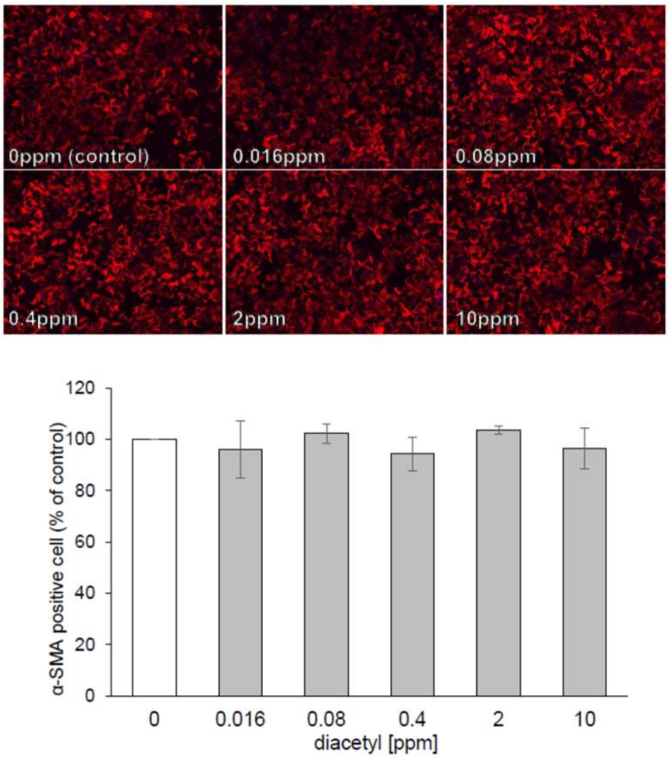

Fig. S2 Effect of diacetyl on HSC activation.

Hepatic stellate cells (HSCs) were isolated from male ddY mice (SLC, Hamamatsu, Japan) by digestion with PRONASE® and collagenase and fractionated by 13% HistoDenz. HSCs isolations were confirmed by autofluorescence derived from vitamin A (ex. 340 nm and em. 550 nm) in which the purity of HSCs was >90%. HSCs were seeded on multi well plates in Dulbecco's modified Eagles medium (DMEM, Sigma) supplemented with 0.1% fetal bovine serum (FBS, Gibco, Life Technologies) and incubated overnight at 37 ° C in a 5% CO_2 atmosphere. When HSCs were treated with diacetyl (0-10 ppm) for seven days, medium containing diacetyl were changed every 2 days. For the immunostaining, HSCs were fixed with 4% paraformaldehyde in PBS (137 mM NaCl, 8.10 mM $Na_2HPO_4 \cdot 12H_2O$, 2.68 mM KCl, and 1.47 mM KH_2PO_4) for 60 min. Cells were permeabilized with 1.0% Triton X-100 in PBS for 60 min and blocked with 3% BSA fraction V (Roche, Basel, Switzerland) for 30 min. HCSs were then incubated with a primary antibody against α-smooth muscle actin (α-SMA; 1:1000, Sigma Aldrich, #A2547) in 1% BSA/PBS overnight at 4° C. Cells were subsequently incubated with goat anti-mouse IgG antibody-conjugated Alexa Fluor 546 (1:1500, Invitrogen, Carlsbad, CA, USA, #A11003) and Hoechst 33342 (1:3000, Dojindo) in 1% BSA/PBS for 60 min at room temperature. Fluorescence microscopy images were obtained and analyzed with a fluorescence microscope (BZ-X800; Keyence, Osaka, Japan). α-SMA-positive cells were determined under fluorescence microscopy, then normalized to the control (100%). Data are expressed as mean±SE (n = 4).
