# Supplementary material for: Non-target GC–MS analyses of fecal VOCs in NASH-hepatocellular carcinoma model STAM mice
Source: Sci Rep. 2023 Jun 1;13:8924. doi: 10.1038/s41598-023-36091-7 (PMC10235110; doi:10.1038/s41598-023-36091-7)
Supplement: Supplementary file 2 — Supplementary Table S1. [file 41598_2023_36091_MOESM2_ESM.pdf]

Supplemental Table 1. List of VOCs on week 4 analyzed by PCA.

RT (min)	Base peak	Name	PC 1 (17.0%)	PC 2 (15.7%)	*p* (unpaired ttest)		
1.2	28		-1.03	-2.82			
1.4	32		-1.94	-2.22			
1.6	14		1.40	-0.79939			
1.6	14		-1.75	2.32			
1.8	40		-0.75	-2.99			
1.8	44		0.55	0.14			
1.9	275		-2.36	0.71			
1.9	17		1.35	-0.20			
2.2	28		0.59	-1.74			
2.3	28		-0.09	-0.72			
2.6	28		0.20	-1.14			
2.8	28		-1.48	-2.55			
2.9	28		1.20	-1.97			
3.0	28		2.80	1.74			
3.1	28		1.09	-1.26			
3.2	28		-0.51	-1.89			
4.0	43	2,3-Butanedione (diacetyl)	3.33	-0.29			
4.3	41	Acetonitrile	0.97	1.62			
5.0	28		-0.18	-1.60			
7.6	18		2.89	-2.38			
12.5	341		-3.56	-1.19			
16.8	281		-1.88	0.33			
18.2	45	2,3-Butanediol	0.71	2.18			
20.7	57	Acetic acid	-2.53	0.42			
