# Supplementary material for: Non-target GC–MS analyses of fecal VOCs in NASH-hepatocellular carcinoma model STAM mice
Source: Sci Rep. 2023 Jun 1;13:8924. doi: 10.1038/s41598-023-36091-7 (PMC10235110; doi:10.1038/s41598-023-36091-7)
Supplement: Supplementary file 3 — Supplementary Table S2. [file 41598_2023_36091_MOESM3_ESM.pdf]

Supplemental Table 2. List of VOCs on week 6 analyzed by PCA.

RT (min)	Base peak	Name	PC 1 (15.1%)	PC 2 (13.0%)	*p* (two-way ANOVA) Diet	STZ	Interact.
1.2	28		-0.69	0.25			
1.5	28		-1.34	-0.68			
1.5	44		-1.53	-0.43			
1.5	32		0.56	-1.48			
1.5	28		0.88	-0.50			
1.6	28		0.76	0.63			
1.6	28		-0.03	1.45			
1.6	14		-1.06	1.38			
1.7	32		0.03	-0.28			
1.7	28		-1.02	1.52			
1.8	43		-0.19	-1.28			
1.9	219		-0.93	1.23			
2.2	28		1.46	-0.44			
2.3	28		0.34	-2.85			
2.4	58	Acetone	0.59	0.17			
2.4	17		2.23	1.29			
2.6	28		1.14	-0.60			
2.7	28		1.14	-0.93			
2.8	28		0.43	0.76			
2.9	28		1.55	0.20			
3.0	28		0.32	0.16			
3.0	28		0.06	-1.47			
3.1	28		2.31	-1.16			
3.2	28		0.46	-3.10			
3.4	45	2-Butanol	-0.12	-1.05			
3.5	45	Isopropyl alcohol	-1.27	-2.46			
3.5	45		-0.61	-1.92			
3.9	43	2-Pentanone	3.49	0.53		3.4E-02	
4.0	43	2,3-Butanedione (diacetyl)	3.68	0.44		3.4E-02	
4.3	281		-1.48	1.96			
5.0	28		1.16	1.47			
8.1	28		-0.10	-1.76			
10.3	94	Methyl-pyrazine	3.45	-1.39		4.3E-02	
10.7	41	Octanal	0.16	-1.03			
12.5	341		0.32	-0.26			
13.4	57	Nonanal	2.33	-2.13			
14.9	18	Acetic acid	3.22	-1.29			
16.6	105	Benzaldehyde	3.84	1.06			
16.8	281		1.51	0.03			
18.9	42	Butyrolactone	3.30	-1.90			4.1E-02
19.4	98	2-Furanmethanol	3.19	0.87			
19.6	73		-0.03	2.65			
19.8	93		2.07	2.46			
20.3	55	2(5H)-Furanone	3.50	-0.39			
20.3	159		1.26	3.77			
20.6	45	Formamide	2.86	-0.85			
21.0	159		1.27	3.76			
21.5	183		0.62	4.05			
22.1	95	1H-pyrrole-2-carboxaldehyde	3.23	-1.30			
22.4	159		1.54	3.11			
