# Supplementary material for: Non-target GC–MS analyses of fecal VOCs in NASH-hepatocellular carcinoma model STAM mice
Source: Sci Rep. 2023 Jun 1;13:8924. doi: 10.1038/s41598-023-36091-7 (PMC10235110; doi:10.1038/s41598-023-36091-7)
Supplement: Supplementary file 4 — Supplementary Table S3. [file 41598_2023_36091_MOESM4_ESM.pdf]

Supplemental Table 3. List of VOCs on week 10 analyzed by PCA.

RT (min)	Base peak	Name	PC 1 (15.8%)	PC 3 (9.9%)	*p* (two-way ANOVA) Diet	STZ	Interact.
0.1	28		0.99	0.53			
1.2	28		0.87	0.34			
1.4	44		-0.79	-2.17			
1.5	28		-0.88	1.23			
1.5	41		-1.02	0.42			
1.6	28		0.16	1.71			
1.6	16		-2.17	1.16			
1.6	14		-2.72	0.12			
1.8	28		-1.55	0.16			
1.8	40		2.00	-0.43			
1.9	17		1.61	-0.68			
1.9	252		0.60	0.76			
2.0	277		-2.05	1.44			
2.1	31		-1.47	2.11			
2.2	28		-0.35	0.34			
2.3	28		1.19	-2.31			
2.3	44		-0.09	-2.55			
2.3	28		0.01	1.87			
2.4	43	Acetone	-3.71	-1.27			
2.6	28		0.46	-1.56			
2.7	28		-0.23	-1.43			
2.8	28		-0.54	-1.14			
2.9	28		1.81	0.20			
2.9	28		0.65	-1.75			
3.0	28		0.40	0.62			
3.1	28		-2.17	-0.91			
3.1	28		-2.67	0.14			
3.1	28		-2.96	0.51			
3.1	28		-1.37	-0.33			
3.2	28		1.29	-2.82			
3.9	43		-2.54	-0.77			
3.9	43	2-Pentanone	-3.32	-0.79			2.3E-03
4.0	43	2,3-Butanedione (diacetyl)	-1.88	0.81			2.3E-02
4.3	41	Acetonitorile	0.53	1.55			
5.0	28		-1.83	-1.07			
5.0	28		-1.51	1.72			
8.1	28		-1.45	1.44			
10.3	94	Methyl-pyrazine	-2.64	-0.67			
10.9	43	3-Methyl-2-butanone	-1.93	0.54		3.6E-02	
11.3	43	1-Hydroxy-2-propanone	-2.29	0.24			2.0E-02
12.5	341		0.14	1.42			
13.4	57	Nonanal	0.25	0.59	4.0E-02		
13.9	122	Trimethyl-pyrazine	-2.26	-1.01			1.3E-02
15.1	45	Acetic acid	0.89	-1.28			
16.6	106	Benzaldehyde	-2.44	-2.50			
16.8	281		0.03	-0.98			
18.9	42	Butyrolactone	-2.79	-0.38			2.7E-02
19.8	119		-2.71	-0.45			
19.8	93		0.70	-2.46			
