# Supplementary material for: Non-target GC–MS analyses of fecal VOCs in NASH-hepatocellular carcinoma model STAM mice
Source: Sci Rep. 2023 Jun 1;13:8924. doi: 10.1038/s41598-023-36091-7 (PMC10235110; doi:10.1038/s41598-023-36091-7)
Supplement: Supplementary file 5 — Supplementary Table S4. [file 41598_2023_36091_MOESM5_ESM.pdf]

Supplemental Table 4. List of VOCs on week 16 analyzed by PCA.

RT (min)	Base peak	Name	PC 1 (17.0%)	PC 4 (8.5%)	*p* (two-way ANOVA) Diet	STZ	Interact.
0.2	28		-0.12	1.29			
1.2	28		0.39	0.48			
1.4	34		1.71	-0.45			
1.4	44		1.72	1.54			
1.5	32		-2.33	1.65			
1.6	32		-2.72	-0.61			
1.6	14		-1.93	-0.58			
1.7	32		-1.71	0.39			
1.8	60		2.32	1.28			
1.8	43		2.04	0.87			
1.9	17		2.79	0.82			
1.9	27		1.16	-1.18			
2.2	28		-0.73	0.31			
2.3	44		-0.70	-2.03			
2.3	28		2.74	-1.82			
2.3	28		-2.72	-0.11			
2.4	41	Butanal	-2.67	-0.91		4.9E-02	
2.5	28		0.83	-1.06			
2.6	28		0.42	-2.18			
2.7	28		0.33	-0.31			
2.8	28		2.93	-0.79			
2.9	28		1.83	0.30			
3.0	28		0.33	0.02			
3.0	28		0.95	-2.71			
3.0	28		-0.82	-0.61			
3.1	41	3-Methyl-butanal	-1.69	-1.85			
3.2	28		-2.03	-0.96			
3.2	28		3.24	-1.33			
3.9	86	2-Pentanone	-2.06	-2.21			
4.0	43	2,3-Butanedione (diacetyl)	-1.32	-0.99			
4.3	41	Acetonitrile	-0.50	-2.87			
4.3	41		1.71	-0.14			
5.0	28		1.98	-0.48			
5.0	28		-1.89	0.52			
8.1	18		-0.03	1.15			
8.9	81	2-Pentyl-furan	1.19	0.80		3.7E-02	
10.4	94	Methyl-Pyrazine	-0.62	-1.72			
10.6	43	3-Methyl-2-butanone	2.27	2.14			
13.3	57	Nonanal	1.84	1.09	4.1E-02		2.0E-02
16.6	105	Benzaldehyde	-0.76	0.53			
19.8	93		-0.41	-0.13			
20.3	55		2.09	0.87			
20.4	161		2.23	-2.55			
20.7	57	Pentacosane	2.33	-0.79			
22.0	94	Phenol	2.48	0.08			
22.4	159		2.25	-2.55			
22.4	107		3.03	-0.59			
22.4	205		0.32	-0.80			
22.4	205		2.59	-0.47			
