# Supplementary material for: Non-target GC–MS analyses of fecal VOCs in NASH-hepatocellular carcinoma model STAM mice
Source: Sci Rep. 2023 Jun 1;13:8924. doi: 10.1038/s41598-023-36091-7 (PMC10235110; doi:10.1038/s41598-023-36091-7)
Supplement: Supplementary file 6 — Supplementary Table S5. [file 41598_2023_36091_MOESM6_ESM.pdf]

Supplemental Table 5. Correlation between fecal diacetyl and tumor incidence at week 16.

Group	Diacetyl [*1]	Tumor incidence			Average of tumor diameter (mm)
		<2 mm	2-4.5 mm	>4.5 mm	
Cont_NFD	2037552	0	0	0	-
	1605427	0	0	0	-
	523322	0	0	0	-
	493069	0	0	0	-
Cont_HFD	657138	0	0	0	-
	4429561	0	0	0	-
	678195	0	0	0	-
	465530	0	0	0	-
	1922024	0	0	0	-
STZ_NFD	12512116	3	0	2	2.83
	1714613	5	0	0	1.60
	1561002	0	0	0	-
	1306371	3	0	3	4.50
	100309	3	0	0	1.00
STZ_HFD	724377	0	1	0	3.00
	50580181	1	0	7	1.75
	29485301	0	1	6	1.43
	14509074	6	3	4	4.62
	631263	0	0	1	4.50
	961689	0	1	2	2.00

[*1] Level of fecal diacetyl detemiend by GC-MS were shown as relative peak areas.
